# Age-Related Trends in the Diet of An Infant’s Cohort in the Northeast of Italy from Six to Twelve Months of Age

**DOI:** 10.3390/nu11020230

**Published:** 2019-01-22

**Authors:** Claudia Carletti, Federica Concina, Paola Pani, Lorenzo Monasta, Alessandra Knowles, Maria Parpinel, Fabio Barbone, Luca Ronfani

**Affiliations:** 1 Clinical Epidemiology and Public Health Research Unit, Institute for Maternal and Child Health-IRCCS “Burlo Garofolo”, via dell’Istria 65/1, 34137 Trieste, Italy; claudiaveronica.carletti@burlo.trieste.it (C.C.); paola.pani@burlo.trieste.it (P.P.); lorenzo.monasta@burlo.trieste.it (L.M.); alessandra.knowles@burlo.trieste.it (A.K.); luca.ronfani@burlo.trieste.it (L.R.); 2 Department of Medicine, University of Udine, via Colugna 50, 33100 Udine, Italy; maria.parpinel@uniud.it; 3 Scientific Direction, Institute for Maternal and Child Health—IRCCS ‘Burlo Garofolo’, via dell’Istria 65/1, 34137 Trieste, Italy; fabio.barbone@burlo.trieste.it

**Keywords:** prospective infant cohort study, age-related trends, commercial baby foods and non-commercial products, energy and nutrients intake

## Abstract

Complementary feeding is recognized as an important predictor of health later in life and is likely to affect the development of food preferences. This paper describes age-related trends in terms of energy, nutrients intake and dietary habits of an Italian infant sub cohort (*n* = 152), enrolled in Trieste. Infant dietary data, collected using a food diary at 6, 9 and 12 months of age, were used to estimate energy and nutrients intake using the Italian food composition database. Age-related trends were calculated using Page’s trend test. An increasing age-trend was observed in the percentages of contribution of macronutrients to total energy intake, with the exception of total lipids, which instead decreased over time. Most of the infants shared a low varied diet especially with regards to protein intake sources, represented mainly by dairy and meat products rather than pulses and fish. This could also account for the low intake of essential fatty acids (ω3) that play an important role in infant neurodevelopment. Moreover, non-commercial baby foods contributed more in terms of quantity, energy and macronutrients intake, compared with commercial products. Healthy eating habits should be encouraged during the first year of life, promoting a varied and well balanced diet at family level.

## 1. Introduction

The transition from exclusive breastfeeding or formula feeding to family foods, which is referred to as complementary feeding (CF), typically covers the period from 6 to 18–24 months of age of infants. This period has been shown to lay the foundations for health and growth [1] and could have an impact on the development of food preferences [2]. During the early years of life, the consumption of health promoting foods, such as fruits and vegetables, reduces the risk of cardiovascular disease, obesity, hypertension, stroke and some cancers in adulthood [3]. Although it has been shown that humans, from birth, tend to prefer sweet, salty and umami tastes over those that are bitter or sour, some studies have suggested that food preferences are acquired through experience. Infants explore new and different flavours from maternal diet, during intra and extra uterine life, through amniotic liquid and breast milk [4]. A varied diet, rich in fruits and vegetables, allows to meet macro and micronutrient requirements and provides adequate and well-distributed energy intake [5]. On the other hand, a CF approach based on the consumption of commercial baby foods may hamper the nutritional education inherent in this learning process, because infants may be less open to accept new flavours that they perceived as unfamiliar [6]. However, extremely few studies exist on the consumption of commercial and home-made baby foods in industrialized countries [7]. Furthermore, only few researches explore infant feeding habits and determine nutrient intakes in detail during the CF period. Among international studies, the most relevant are four large cohort studies: the Norwegian Mother and Child Cohort Study (MoBa) [8], the Dortmund Nutritional and Anthropometric Longitudinally Designed Study (DONALD) [9], the Avon Longitudinal Study of Pregnancy and Childhood (ALSPAC) [10] and the Public health impact of long-term, low-level mixed element exposure in susceptible population strata study (PHIME) [11]. However, all of these studies investigate how dietary habits combined with genetic and/or environmental aspects, influence the health and development of children. Moreover, very few data are available at national level [12,13].

The aim of this paper is to describe age-related trends in energy, macro and micronutrients intake and Body Mass Index (BMI), during the first year of life with a special focus on whether the dietary habits of this sub cohort privileged home-made versus commercial baby foods. The present study offers the opportunity to better understand how eating patterns change during this sensitive time and to identify critical aspects of feeding practices during CF, which should be monitored closely and addressed by recommendations, targeted to parents.

## 2. Materials and Methods

We investigated age-related trends in nutritional and anthropometric data obtained from an ongoing prospective infant cohort study conducted in the north east of Italy (Trieste Infant Food cohort—TIF cohort). In this study, detailed data were collected from 400 healthy infants recruited at birth at the Institute for Maternal and Child Health—IRCCS “Burlo Garofolo” Trieste, between July 2007 and July 2008. The study, which started in 2006 as a part of the PHIME European research project [14], was conducted in accordance with the Declaration of Helsinki and the protocol was approved by the ethics committee of the Institute. All participating subjects gave their informed consent for inclusion, before they were enrolled in the study. The study design, methods and sampling procedures have already been reported [15].

In brief, infants were eligible if their birth weight was 2000 g or more, gestational age was 36 completed weeks or more, if they were not affected by severe diseases or congenital malformations that required hospital admission and if their mothers were resident in the province of Trieste. Dietary data were collected using a 3-day dietary (3-DD) record (food diary) provided to mothers during the first contact, with instructions on how to record type, quantity and method of feeding over a 24 h period on three separate non-consecutive days, including one at the weekend at 3, 6, 9, 12, 18, 24 and 36 months of age of the infants. Mothers were asked to report weight and length of their children as measured by their community paediatrician during periodic health checks at 1, 3, 5–6, 8, 12, 18, 24 and 36 months of age. In addition, demographic, educational, social and anthropometric data of the mothers and fathers were obtained from a validated questionnaire designed for the PHIME study [14].

The present paper focuses on only 3 (6, 9, 12 months) of the 7 collection points, because this is the period in which CF starts and the changes in the infant’s diet are greater and most rapid [16]. Only subjects who completed the food diary at all three collection points were considered for data analysis (TIF sub cohort). The complete nutritional analysis of our cohort (TIF cohort) at the same collection points considered in this paper has been fully described in a previous paper [17].

Data extracted from food diaries were analysed using the Microdiet V2.8.6. software (Microdiet software—Downlee Systems Ltd., High Peak, UK), which contains the Italian Food Composition database for Epidemiological Studies [18], integrated with nutritional data from food labels in the case of commercial products and formula milk and from literature for human milk [19,20]. Full details of the methodology are published elsewhere [21].

The nutritional analysis was performed on 28 food components: proteins (total), carbohydrates (available and soluble, starch, fibre), lipids (total, saturated, monounsaturated and polyunsaturated fatty acids; oleic, linoleic and linolenic acid; cholesterol), minerals (sodium, calcium, potassium, iron, zinc) and vitamins (vitamin B1, vitamin B2, vitamin B6, vitamin C, vitamin D, vitamin E α-TE, retinol, retinol eq., niacin, folate).

Foods were classified into 21 food groups based on the methodology proposed by Talamini et al. (2006) [22] and modified as follows: cereals, milk and dairy products, eggs, vegetables, fruit, seeds and nuts, herbs and spices (also salt), fish, meat, fats and oils, beverages (water and beverage without sugar), sugars, sweets and desserts, soft drinks, tubers, sauces, cured meats.

Based on the methodology used by Noble and colleagues in the ALSPAC study [23], we also classified foods into 4 food types: commercial products (CP; data from food labels), non-commercial products (NCP; data already present in the Italian food composition database), breast-feeding (BF) and formula feeding (FF). Slight methodological differences did exist between the two classifications, since our study did not consider rusks and fruit juices/beverages as separate categories from the other CP [23].

For the analysis of CF practices and of the contribution of each food type in terms of energy and macronutrients intake, we considered three separate categories, two relating to complementary feeding (NCP and CP) and one to milk based feeding (BF plus FF).

Furthermore, in order to highlight the age-related trends in daily intake of different food groups, we performed the analysis on the food groups that contributed the most to macronutrients intake: cereals, milk and dairy products, eggs, fruit and vegetables, pulses, fish and meat and cured meat.

Categorical data are presented as absolute frequencies and percentages, while continuous data as medians and interquartile ranges (IQR: 1st-3rd quartiles). The mean daily intake of macro-, micronutrients and energy was calculated, for each infant, on a 3-day observation basis, excluding the use of supplements.

Differences in continuous variables were assessed using non-parametric rank-sum Mann–Whitney tests or signed-rank Wilcoxon test as appropriate, while differences between categorical variables were analysed with two-tailed Fisher exact tests. Trend analysis was performed using Page’s trend test. With regards to the repeated measures (quantities of nutrients at 6, 9 and 12 months for the same subjects), the signed-rank test by Wilcoxon allowed us to test the significance of the difference between measures at different time points (i.e. 6 months vs. 9 months and 9 months vs. 12 months). Page’s trend test, instead, was used to verify if there was a significant trend (decreasing or increasing) taking into account the three measures simultaneously. Both tests (Wilcoxon’s and Page’s) are non-parametric rank tests, not imposing hypothesis on the distribution of the data analysed and on their residuals. Statistical significance for all tests was set at a *p*-value below 0.05.

Anthropometric measures were compared with the WHO growth standards using WHO Anthro software and are reported as BMI *z*-scores [24,25].

All the analyses were carried out using Stata/IC 14.2 (StataCorp LLC, College Station, TX, USA).

## 3. Results

The number of food diaries available were 268 at 6 months, 179 at 9 months and 176 at 12 months of age but only for 152 infants completed food diaries were available at each collection points. The characteristics of the mothers and infants of the TIF sub cohort (*n* = 152) are described in Table 1. The differences in sample size reported in the table for some variables are due to missing data in the questionnaires.

Forty-five percent of mothers were 35 years old or more (mean age 34.4 ± 4.2), 92% were born in Italy, 91% were married or living with the father of the child, 88% had a medium-high level of education, 96% were in employment and 68% had a pre-pregnancy BMI that fell within the normal range. Most infants (94%) were born between 38 and 42 weeks of gestation, 83% by vaginal delivery. Infants were equally distributed by sex: 78 (52%) males and 73 (48%) females. The majority (94%) weighed between 2500 g and 4199 g at birth (mean weight 3432 ± 442 g) and 83% were between 46.0 and 52.9 cm long (mean length 50.5 ± 1.9 cm).

A comparison between the mothers belonging to the TIF sub cohort (*n* = 152) and the remaining TIF group (*n* = 248) shown a statistically significant difference in terms of level of education (*p* = 0.001), nationality (*p* = 0.041) and mean age at delivery (*p* = 0.004). More specifically, mothers belonging to the TIF sub cohort had a higher level of education (88% vs. 79%), were more likely to be Italian (92 vs. 85%) and had higher mean age at delivery (34.4 ± 4.2 vs. 33.0 ± 4.9), as compared with the remaining TIF cohort.

At 6 months of age, 91% of infants were already on complementary feeding, with 63% of these still being breastfed, while only 9% were still exclusively breastfed. At 9 months of age, 99% of infants were receiving complementary foods, alone (7%) or in combination with breast milk (56%) or infant formula (36%) and only one infant (1%) was still exclusively breastfed. At 12 months of age, 39% of infants were still breastfeeding. A complete overview of the feeding practices in the TIF cohort, during the first 24 months of life of the infants is published elsewhere [15].

Daily energy, macro- and micronutrients intake are shown in Table 2.

The intake of energy, total proteins, saturated fatty acids, starch, calcium, potassium, vitamin B2, vitamin B6 and vitamin E α-TE significantly increased between 6 and 12 months of age. In particular total proteins intake almost tripled, from 13.0 (8.9; 17.0) g/day at 6 months to 31.9 (25.1; 9.4) g/day at 12 months, while the intake of calcium and potassium almost doubled, from 313.5 (228.4; 467.9) mg/day and 610.3 (466.9; 829.5) mg/day at 6 months to 587.1 (416.1; 773.6) mg/day and 1232.7 (912.6; 1621.2) mg/day at 12 months of age, respectively.

Although the trend analysis shown a statistically significant increase in the intake of total lipids, monounsaturated fatty acids, oleic acid, cholesterol, available carbohydrates, fibre, iron, sodium, zinc, vitamin B1, retinol, retinol eq, niacin and folate between the ages of 6 and 12 months, this trend flattened between 9 and 12 months. The only exception was zinc intake for which no statistically significant difference was found between 6 and 9 months of age. Moreover, as shown in Table 2, there was a significant decrease in linoleic and linolenic acids intake, with a non-statistically significant difference, between the ages of 6 and 9 months for linoleic acid and 9 and 12 months for linolenic acid. Finally, no age-related trend was observed regarding polyunsaturated fatty acids, soluble carbohydrates, vitamin C and vitamin D intake, despite there being a statistically significant difference between consecutive collection points. This was not true for vitamin D when intakes at 6 and 9 months were compared.

Overall, NCP contributed more, in terms of energy and macronutrients, compared to CP at each of the three collection points, with the exception of available carbohydrates at 6 months which were mainly from CP. In particular, for NCP consumption, the trend analysis revealed a statistically significant increase in energy and nutrients intake between the ages of 6 and 12 months (*p* < 0.001). Moreover, there was a statistically significant increase in CP consumption from 6 to 9 months (*p* < 0.001) followed by a statistically significant decrease between 9 and 12 months (*p* < 0.001). The only exceptions were soluble carbohydrates which continued to increase (*p* < 0.001), available carbohydrates and proteins which showed a non-significant decrease between 9 and 12 months of age (*p* = 0.0787). At 6 months, soluble carbohydrates were found not to contribute to energy intake from CP (0%E) but this was probably due to missing data in baby food labels [21].

The age-related trends in daily intake of the selected food groups are reported in Table 3.

**Figure 1:**
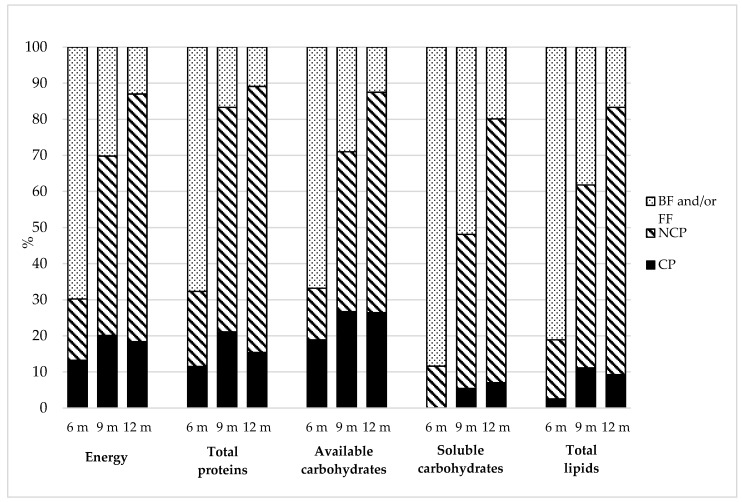
Contribution of different food types in terms of energy and macronutrients intake. Abbreviation: BF = Breastfeeding; FF = Formula feeding; NCP = Non-commercial products; CP = Commercial products; m = months of age.

There was a statistically significant increase in the consumption of all food groups between the ages of 6 and 12 months, with the exception of milk and dairy products. The consumption of this food group decreased significantly, presumably as a result of the fact that, as reported in Figure 1, the consumption of breast milk and infant formula reduced between the ages of 6 and 12 months. The infants’ diet was based on (in decreasing order of consumption): milk and dairy products (from 568.4 g/day at 6 months to 392.4 g/day at 12 months), fruits and vegetables (from 107.7 g/day at 6 months to 173.8 g/day at 12 months), cereals (from 8.3 g/day at 6 months to 45.6 g/day at 12 months), beef and cured meet (from 0 g/day at 6 months to 36.6 g/day at 12 months). The consumption of eggs (0 g/day at all three collection points), pulses (from 0 g/day at 6 months to 4.6 g/day at 12 months) and fish (from 0 g/day at 6 months to 3.0 g/day at 12 months) was negligible.

BMI *z*-score distribution at 6, 9 and 12 months of age is shown in Table 4. Anthropometric data were available only for 115 (76%) infants at 6 months and 117 (77%) infants at 9 and 12 months. Only one infant (1%) classified as overweight according to WHO Standards [24,25].

## 4. Discussion

Our results show that, in the first year of life, the diet of the TIF sub cohort infants was characterized by low variety, excessive intake of proteins, mainly from animal sources and saturated fatty acids and low intake of essential fatty acids (ω-3). This can lead to negative short and long-term health consequences, as already reported in a previous paper on the same cohort [17].

The assessment of the dietary intake of infants during the first year of life is generally recognized as difficult to accomplish. Only few national and international studies have analysed the dietary habits of infants in a period that is so crucial for child growth, development and health and is characterized by the transition from exclusive milk feeding to family foods. Among the few available studies, one of the most important is the DONALD study, a prospective cohort study conducted by the University of Bonn, Germany, to assess the relationship between diet, nutrition and development during childhood and adolescence [9]. In particular, Foterek et al. (2016) reported significant time and age trends of energy and macronutrients intake between the ages of 3 months and 3 years in the DONALD cohort. With regards to the first year of life in particular, they observed a decrease in total lipids intake and a compensatory increase in total proteins and available carbohydrates intake [26]. The same trend in macronutrients intake was also observed in the TIF sub cohort, with energy intake from total proteins increasing significantly from 10% E at 6 months to 16% E at 12 months (Table 2). Our results also reveal that, in contrast with national and international recommendations, total protein intake derived mainly from animal sources (dairy products and meat/cured meat). This was reflected in the significant increase in calcium and vitamin B intake which mainly relies on animal food sources. Substantiating the hypothesis of low food variety in our sub cohort, at 12 months of age only few infants were consuming pulses, eggs and fish and, even then, only in small quantities (Table 3) [2,27]. The introduction of these food groups after 9 months of age may also explain the non-statistically significant difference observed in the intake of polyunsaturated fatty acids, oleic and linoleic acids, cholesterol and zinc between the ages of 6 and 9 months.

As shown in Table 2, there was a significant trend increase in saturated fatty acids intake and a significant trend decrease for linoleic and linolenic acids. This is probably due the combination of reduced breast and/or formula feeding and low consumption of fish, which are the main sources of polyunsaturated fatty acids and increased consumption of meat/cured meat and dairy products, which are rich in saturated fatty acids (Table 3).

Two studies have analysed the contribution of CP and NCP in terms of energy and macronutrients intake [7,28]. In the first study, the authors found significant differences between the two food types in terms of energy and macro and micronutrients content but the sizes of the effect were small throughout the study period and therefore scarcely relevant for dietary practice [7]. The second study showed that an infants’ diet based mainly on NCP is associated with a more varied diet during the first year of life and with reduced adiposity [28]. Unfortunately, these results cannot be compared with ours because of the different criteria used to classify the categories in question. Hilbig and colleagues estimated the energy and nutrients content of CP and NCP meals instead of single CP and NCP foods, as in our classification. We observed that the consumption of NCP was prevalent in our infants’ diet at all three collection points. In case of CP, only soluble carbohydrates displayed a significant trend increase from 0% (0 g/day) of total soluble carbohydrates intake at 6 months to 7% (3.2 g/day) at 12 months, probably due to increased consumption of sweets and/or desserts and sweet beverages. Also, the intake of soluble carbohydrates from NCP increased from 12% (5.4 g/day) of total soluble carbohydrates intake at 6 months to 73% (33.2 g/day) at 12 months, however, this percentage includes the contribution of homemade desserts and sweets and fruits (Figure 1).

Since, only one infant in the TIF sub cohort classified as overweight, we decided not to study the influence of different complementary feeding habits (i.e., prevalent use of CP vs. NCP) on infant BMI, as reported in another study [28]. As reported by other authors, evidence regarding the influence of CF choices (type of foods and timing of introduction) on growth and body composition remain inconclusive [28,29].

The strengths and limitations of our study need to be discussed. Among the major strengths are the frequent and detailed measurements of nutrition and growth, the adoption of a rigorous design (prospective cohort study) that allowed for accurate data collection, the use of a 3-day dietary (3-DD) record which is the gold standard for the assessment of dietary intake during infancy [30], the extraction of food composition and nutritional data using an opportunely integrated version of the Microdiet software. However, some limitations must be considered when interpreting the results. First, the widespread use of commercial baby foods, generally characterized by poor nutritional labelling, may have limited the precision of the analysis with regards to the intake of micronutrients such as vitamins and some minerals. Instead, the data on energy components (total proteins, available carbohydrates and total lipids) can safely be considered complete [21]. Second, the method employed to assess the consumption of breast milk (frequency of feeds, perceived length of each feed by mothers) and the use of a literature-derived composition that does not take inter- and intrasubject variability into consideration, may have led to underestimation of nutrients intake. Nevertheless, we decided not to use the double weighing methodology, which is considered the most rigorous method, because we feel it is an invasive procedure that goes against the main principle of breastfeeding. The methodology we adopted in this study has been used before in other international infants cohort studies [8,10]. Finally, the results shown that a certain degree of auto-selection may have occurred among mother/child pairs who completed the food diaries at all three collection points, compared to the rest of the TIF cohort, because of the food diaries being quite lengthy and complex. That a bias might be present is suggested by the higher duration of breastfeeding (mean 12.3 ± 8.6 months) and higher prevalence of breastfeeding at 12 months of age (39%), compared to the rates reported in previous regional and national surveys [31]. This auto-selection may have affected the rate of reduction of the sampling size by 62% (152/400). However, as already reported elsewhere, the comparison between the mothers of the TIF sub cohort and the rest of the TIF group shows they shared a similar approach to infant feeding practices [17].

## 5. Conclusions

The data from our study demonstrate an increasing age-trend in the percentages of contribution of the main macronutrients to total energy intake, except for total lipids, during the first year of life. Most of the infants shared a low varied diet, as already reported in a previous paper [32], with energy intake from total proteins deriving mainly from selected animal sources (dairy products and meat/cured meat). This might be reflected in the unbalanced intake of fatty acids, also as a consequence of reduced breast/formula feeding, that favours saturated over polyunsaturated fatty acids that play an important role in infant neurodevelopment.

Overall, NCP contributed more, in terms of energy and macronutrients intake, compared to CP, during the CF period. However, there was a linear increase in CP consumption between 6 and 9 months of age.

Our results suggest that, in order to promote healthy eating habits during the first year of life and encourage a varied and well-balanced diet at family level, a number of public health strategies should be implemented at national level. Among these the protection, promotion and support of breastfeeding during the first year of life and the development of evidence-based guidelines on infant feeding, endorsed by all health professionals and free from commercial interests.

## Figures and Tables

**Table 1:** Characteristics of mothers at enrolment and children at birth (*n* = 152).

Characteristic	*n*	%
**Infant sex** (*n* = 151)		
Male	78	52
Female	73	48
**Birth weight** (*n* = 151)	
<2500 g	0	0
2500–4199 g	142	94
≥4200 g	9	6
**Birth length** (*n* = 150)		
<46 cm	1	1
46–52.9 cm	125	83
≥53 cm	24	16
**Birth maternal age** (*n* = 152)		
<30 years	26	17
30–34 years	57	38
≥35 years	69	45
**Maternal nationality** (*n* = 152)		
Italian	140	92
Foreigner	12	8
**Maternal marital status** (*n* = 152)		
Married/living with partner	138	91
Separated/divorced	5	3
Single/not living with partner	9	6
**Maternal education** (*n* = 151)		
≤Secondary school	18	12
Completed high school or equivalent	58	38
Bachelor’s degree or higher	75	50
**Employment** (*n* = 137)		
Yes	131	96
No	6	4
**Maternal pre-pregnancy BMI** (*n* = 151)		
<18.5	14	9
18.5–25	102	68
25–30	25	17
≥30	10	7
**Gestational age at birth** (*n* = 150)		
36–37 weeks	9	6
38–42 weeks	141	94
**Type of birth** (*n* = 151)	
Vaginal	125	83
Caesarean	26	17

Abbreviation: *n* = number of subjects; % = percentage of subjects.

**Table 2:** Daily energy, macronutrient and micronutrients intake from 6- to 12 months of age (*n* = 152)

Nutrient	6 Months	9 Months	12 Months	*p*-Value 6 vs. 9	*p*-Value 9 vs. 12	*p*-Value Trend
	Median (IQR)	Median (IQR)	Median (IQR)			
Energy (kJ)	2246.0 (1881.5; 2808.7)	3254.7 (2619.6; 3631.3)	3274.0 (2851.8; 3825.0)	<0.001	0.0357	<0.001↑
Total proteins (g)	13.0 (8.9; 17.0)	27.0 (21.5; 33.7)	31.9 (25.1; 39.4)	<0.001	<0.001	<0.001↑
*Total proteins (%E)*	*9.7*	*13.9*	*16.3*			
Total lipids (g)	23.7 (20.0; 29.1)	29.8 (23.8; 36.8)	29.3 (24.1; 35.6)	<0.001	0.7171	<0.001↑
*Total lipids (%E)*	*39.7*	*34.5*	*33.7*			
Saturated fatty acids (g)	8.9 (7.6; 10.5)	10.3 (8.3; 12.4)	11.3 (8.7; 15.3)	<0.001	<0.001	<0.001↑
Monounsaturated fatty acids (g)	9.0 (7.5; 11.2)	10.6 (8.4; 13.9)	11.4 (8.1; 13.4)	<0.001	0.9121	0.0058↑
Oleic acid (g)	8.6 (7.3; 10.9)	10.1 (7.8; 13.3)	10.1 (7.3; 12.2)	0.0014	0.7461	0.0224↑
Polyunsaturated fatty acids (g)	2.9 (2.1; 4.0)	3.2 (2.5; 4.3)	2.7 (2.0; 3.7)	0.0183	<0.001	0.8965
Linoleic acid (g)	2.4 (1.8; 3.3)	2.6 (1.9; 3.5)	2.0 (1.5; 3.0)	0.2110	<0.001	0.0054↓
Linolenic acid (g)	0.8 (0.6; 1.1)	0.4 (0.3; 0.6)	0.4 (0.3; 0.6)	<0.001	0.7336	<0.001↓
Cholesterol (mg)	68.3 (21.0; 96.2)	63.2 (35.4; 91.8)	88.1 (62.9; 118.4)	0.4178	<0.001	<0.001↑
Available carbohydrates (g)	67.0 (54.6; 87.6)	98.3 (82.6; 117.8)	103.6 (84.8; 128.0)	<0.001	0.0960	<0.001↑
*Available carbohydrates (%E)*	*49.9*	*50.5*	*53*			
Soluble carbohydrates (g)	46.5 (38.2; 54.7)	42.9 (33.3; 52.1)	45.4 (37.5; 53.7)	<0.001	0.0222	0.8352
*Soluble carbohydrates (%E)*	*32.5*	*20.7*	*21.8*			
Starch (g)	7.3 (0.7; 14.7)	24.9 (15.8; 40.1)	32.6 (18.3; 49.7)	<0.001	<0.001	<0.001↑
Fibre (g)	2.8 (1.0; 5.4)	6.8 (4.5; 9.0)	6.8 (4.7; 8.7)	<0.001	0.4290	<0.001↑
Iron (mg)	2.1 (0.6; 5.6)	4.9 (2.8; 7.3)	4.6 (3.2; 6.1)	<0.001	0.1343	<0.001↑
Calcium (mg)	313.5 (228.4; 467.9)	526.7 (367.0; 678.8)	587.1 (416.1; 773.6)	<0.001	<0.001	<0.001↑
Sodium (mg)	232.9 (167.2; 464.4)	517.1 (371.1; 865.2)	658.3 (422.9; 896.1)	<0.001	0.0632	<0.001↑
Potassium (mg)	610.3 (466.9; 829.5)	1007.3 (731.7; 1356.4)	1232.7 (912.6; 1621.2)	<0.001	<0.001	<0.001↑
Zinc (mg)	3.1 (2.5; 4.1)	3.5 (2.2; 4.6)	4.0 (3.1; 4.9)	0.3215	<0.001	<0.001↑
Vitamin B1 (mg)	0.4 (0.2; 0.6)	0.6 (0.4; 0.8)	0.6 (0.4; 0.7)	<0.001	0.5061	<0.001↑
Vitamin B2 (mg)	0.5 (0.3; 0.9)	0.8 (0.5; 1.0)	0.9 (0.6; 1.2)	<0.001	<0.001	<0.001↑
Vitamin B6 (mg)	0.4 (0.1; 0.6)	0.8 (0.6; 1.0)	0.9 (0.7; 1.2)	<0.001	<0.001	<0.001↑
Vitamin C (mg)	52.1 (35.5; 84.0)	63.0 (45.6; 88.9)	50.8 (36.2; 70.1)	<0.001	<0.001	0.7720
Vitamin D (µg)	0.3 (0.2; 5.2)	0.7 (0.2; 4.3)	0.5 (0.2; 1.4)	0.6233	0.0017	0.7890
Vitamin E α-TE (mg)	0.9 (0.2; 1.7)	2.5 (1.7; 3.7)	2.7 (1.7; 3.9)	<0.001	0.0470	<0.001↑
Retinol (µg)	10.3 (0; 117.4)	49.0 (25.5; 95.8)	108.0 (65.4; 190.9)	0.1219	<0.001	<0.001↑
Retinol eq (µg)	100.3 (6.2; 318.6)	422.8 (223.0; 730.4)	460.7 (274.9; 680.9)	<0.001	0.5950	<0.001↑
Niacin (mg)	2.9 (1.5; 5.5)	6.0 (4.0; 8.2)	6.1 (4.6; 8.5)	<0.001	0.1416	<0.001↑
Folate (µg)	81.3 (63.4; 115.6)	111.1 (80.6; 145.6)	105.6 (84.6; 142.4)	<0.001	0.1008	<0.001↑

Significant differences between 6 and 9 months and 9 and 12 months were tested using Wilcoxon signed-rank test (*p* < 0.05). Trends (↑ increasing or ↓ decreasing) were tested using Page’s trend test (*p* < 0.05).

**Table 3:** Age-related trends in daily food group intake (g/day) between 6 and 12 months of age (*n* = 152).

	6 Months	9 Months	12 Months			
Food Group	Median (IQR)	Median (IQR)	Median (IQR)	*p*-Value 6 vs. 9	*p*-Value 9 vs. 12	*p*-Value Trend
Cereals (g)	8.3 (0; 23.3)	31.8 (18.5; 47.6)	45.6 (28.9; 69.5)	<0.001	<0.001	<0.001↑
Milk and dairy products (g)	568.4 (446.7; 697.0)	416.1 (319.3; 530.9)	392.4 (309.9; 516.4)	<0.001	0.3596	<0.001↓
Eggs (g)	0 (0; 0)	0 (0; 0)	0 (0; 0.8)	0.0024	<0.001	<0.001↑
Fruits and vegetables (g)	107.7 (32.5; 193.2)	170.0 (83.4; 253.8)	173.8 (120.6; 276.4)	<0.001	0.0835	<0.001↑
Pulses (g)	0 (0; 0)	3.2 (0; 14.6)	4.6 (0; 16.6)	<0.001	0.5114	<0.001↑
Fish (g)	0 (0; 0)	0 (0; 26.7)	3.0 (0; 26.7)	<0.001	0.6556	<0.001↑
Meat and cured meat (g)	0 (0; 22.8)	28.3 (10.2; 53.3)	36.6 (15.6; 53.3)	<0.001	0.2014	<0.001↑

Abbreviation: IQR = InterQuartile Range. Significant differences between 6 and 9 months and 9 and 12 months were tested using Wilcoxon signed-rank test (*p* < 0.05). Trends (↑ increasing or ↓ decreasing) were tested using Page’s trend test (*p* < 0.05).

**Table 4:** BMI *z*-scores distribution at 6, 9 and 12 months of age (*n* = 115 at 6 months; *n* = 117 at 9 and 12 months).

	6 Months	9 Months	12 Months
	*n* (%)	*n* (%)	*n* (%)
−2SD	8 (7)	1 (1)	-
−1SD	18 (17)	14 (12)	9 (8)
0SD	79 (69)	88 (75)	93 (79)
+1SD	9 (8)	13 (11)	14 (12)
+2SD	1 (1)	1 (1)	1 (1)

Abbreviation: *n* = number of subjects; % = percentage of subjects; SD = Standard Deviation.
